# Association of serum vitamin D level with age in individuals with normal renal function

**Published:** 2012-01-05

**Authors:** Hamid Nasri, Mohammad-Reza Ardalan

**Affiliations:** ^1^Department of Internal Medicine, Shahrekord University of Medical Sciences, Iran; ^2^Department of Internal Medicine, Tabriz University of Medical Sciences, Tabriz, Iran

**Keywords:** Vitamin D deficiency, Age, Vitamin D level

## Abstract

There is no sufficient data on the relation between vitamin D status and age in subjects with normal renal function. The aim of this study was to investigate the association of serum 25-hydroxy vitamin D (25[OH]D) concentrations with age of Iranian healthy individuals. The data were collected from 259 ambulant healthy medical staffs who had the inclusion criteria of the study. Fasting 25-hydroxy vitamin D level was measured. Vitamin D deficiency was defined as having a 25(OH)D concentration <25 nmol/L. The average of persons ages were 20–65 years with mean age of 34±9 years, and about 57.5% of the participants were female. The mean±SD vitamin D level of the subjects was 29±16 nmol/L (median=26 nmol/L). Also 48% of the individuals had vitamin D deficiency. In this study, there was no association between vitamin D level and gender of the participants. A significant positive correlation of age with vitamin D level was found (r =0.002). It seems that for evaluation of 25-OH-vit D status, age should be noticed.

**Implication for health policy/practice/research/medical education:**

In a study on 259 ambulant healthy medical staffs with age of 20–65 years with mean age of 34±9 years, a significant positive correlation of age with vitamin D level was found (r =0.002). It seems that for evaluation of vitamin D level status, age should be noticed.

## Introduction

The circulating level of 25(OH)D is normally used as the indicator of an individual’s vitamin D level. This indicates the quantity of vitamin D generated in the skin during ultraviolet B (UVB) exposure (cholecalciferol (25(OH)D_3_)) and the quantity of vitamin D ingested from food [ergocalciferol (25(OH)D_2_)](1). Vitamin D [25(OH)D] is necessary for normal growth and development. Vitamin D deficiency is common in as many as one half of middle-aged to elderly subjects in developed countries (1,2). Vitamin D deficiency is not sufficiently evaluated in older men and, there is no sufficient data on the relation between vitamin D level and age. The aim of this study was to investigate the association of serum 25(OH)D concentrations with age of a group of healthy Iranian subjects.

## Subjects & Methods

### Participants

Data from 259 ambulant medical staff adults, students and other subjects who had the inclusion criteria of the study were collected for this study. Study was conducted in 2010. All participants (aged 18–65 years) were Iranian origin and from a central province of the country, without having any organic problem (i.e. kidney failure or the presence of diabetes mellitus) or taking the drugs, which affect calcium homeostasis. Other exclusion criteria were absence of pregnancy or lactation, no presence of convulsion or its history. Individuals who were currently under calcium, vitamin D or any other supplements, or using weight loss agents, participating in commercial weight loss programmes, and a desire to lose weight were also excluded from the investigation.

### Laboratory assays

A fasting blood sample (10 ml) was drawn from each subject. Then samples were centrifuged and serum were extracted. Serum samples were obtained in the morning after an overnight fasting, and were frozen at −80 °C. Analyses of the samples were done in one laboratory. EIA Kits (Immunodiagnostic systems Company, USA) technique was used in order to determine 25-hydroxy vitamin D levels. The tests were done with stat fax 2100 (Awareness; USA). Renal function tests consisting serum creatinine and blood urea nitrogen (BUN) were also measured. For the present analysis, vitamin D deficiency was defined as having a 25(OH)D concentrations <25 nmol/L (3).

### Ethical approval

Information sheets and consent forms were distributed between the participant and all the subjects signed consent forms. Research study was approved by the ethics committee of Shahrekord University of Medical Sciences, Iran.

### Statistical analysis

Statistical analysis was performed by SPSS 11 (SPSS Inc., Chicago, USA). Statistical correlations were performed using Pearson correlation coefficients to assess simple associations between continuous variables. In addition, partial correlation test was used to exclude confounding factors. The independent t-test was used to compare vitamin D and BMI for male and female subjects. *P*≤0.05 was considered statistically significant.

## Results

**Figure 1:**
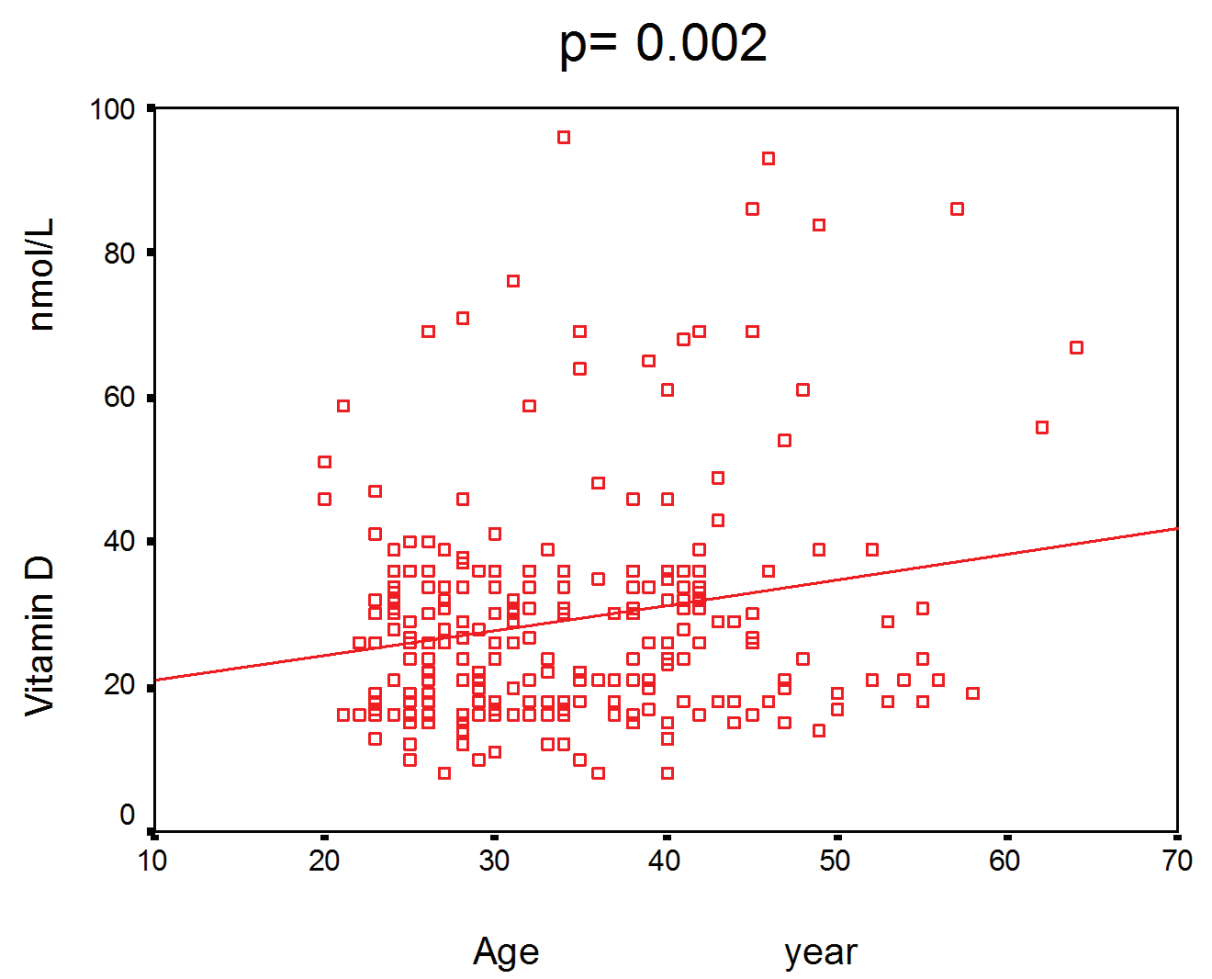
Significant association of age with vitamin D level

Participants age ranged from 20 to 65 years with mean±SD age of 34±9 years, 57.5% of the participants were female. The mean±SD vitamin D level of the participants was 29± 16 nmol/L (median= 26 nmol/L). The characteristics of the study samples are shown in Table 1. About 48% of the subjects had vitamin D deficiency (serum 25(OH)D concentrations below 25 nmol/L). In this study, there was not any correlation between vitamin D level and gender of the participants (p>0.05). A significant association of age with vitamin D level was seen (r= 0.002; Figure 1).

**Table 1:** Demographic characteristics of the subjects

Participant characteristics	Mean ± SD	Minimum	Maximum
Vitamin D level (nmol/L)	29.0 ± 16.0	8.0	96.0
Age (years)	34.0 ± 9.0	20.0	64.0
Weight (kg)	68.8 ± 12.0	43.0	120.0

## Discussion

The aim of our study was to examine the prevalence of vitamin D deficiency and identify risk factors for its occurrence. In the present study, we found that 48% of our participants had vitamin D deficiency. A significant correlation of age and vitamin D level was existed. Vitamin D has captured notice as an important factor of bone health, but there is no common definition of optimal vitamin D status. In contrast to our findings, Orwoll *et al*. conducted a study on 1606 older men in the general community. They found that vitamin D deficiency was common in older men and was especially prevalent in obese, sedentary men living at higher latitudes (4). Similarly, Linnebur *et al*. conducted a study on eighty patients. They found that fifty-nine (74%) had vitamin D insufficiency. They observed that vitamin D insufficiency is prevalent in ambulatory, and especially obese, elderly living in Denver, Colorado, despite vitamin D intake consistent with national recommendations (5).

Vitamin D deficiency has been reported as a risk factor for various diseases like osteoporosis, poor controlling of diabetes and some types of cancer (6). Likewise to our study, Bischof *et al*. conducted a study on the samples of 483 adults. They found positive association of ages with 25-OH-vit D (7). Similar to our finding, Masoumpour *et al*. observed that serum level of 25-hydroxyvitamin D did not decline with age. Their study was carried out in center of Iran, and was consisted of 520 men with a mean±SD age of 45±15 years and a mean±SD 25-hydroxyvitamin D level of 35 ± 17 nmol/L. They found that more than 33.9% of men had a low level of 25-hydroxyvitamin D (≤25 nmol/L) (8). In contrast to these results, Smotkin-Tangorra *et al*. concluded that vitamin D insufficiency was associated with increased age. In their study, 217 obese subjects were tested and severely low vitamin D levels were observed in 21.6% of the patients (9).

## Conclusion

It seems that for evaluation of 25-OH-vit D status, age should be noticed.

## Authors’ contributions

HN and MRA wrote the manuscript equally.

## Conflict of interests

The authors declared no competing interests.

## Ethical considerations

The research followed the tenets of the declaration of Helsinki; written informed consent was obtained and the research was approved by ethical committee of Shahrekord University of Medical Sciences.

## Funding/Support

This study was supported by a grant from Shahrekord University of Medical Sciences, Iran. This study extracted from a MD thesis.
